# ASO Author Reflections: The Role of Neoadjuvant Chemotherapy in Invasive Intraductal Mucinous Neoplasms of the Pancreas

**DOI:** 10.1245/s10434-024-15002-8

**Published:** 2024-03-14

**Authors:** A. Fogliati, Mark J. Truty

**Affiliations:** 1 Division of Hepatobiliary and Pancreas Surgery, Mayo Clinic, Rochester, MN USA; 2 Department of Medicine and Surgery, University of Milano Bicocca, Milano, Italy

## Past

Intraductal mucinous cystic neoplasms (IPMN) of the pancreas are relatively common,^1^ and these cystic tumors have potential for progression into pancreatic ductal adenocarcinoma (PDAC), though their prognosis is debated to be less severe than that of de novo PDAC. The management of IPMN was originally addressed by the first international consensus guidelines in 2006,^2^ which were later revised in 2012 and 2017.^3^ However, these IPMN guidelines mostly focus on risks of an invasive component and the surgical indications and their follow-up, without mentioning the possible role of neoadjuvant chemotherapy in IPMNs whose invasive nature has been pathologically demonstrated preoperatively. Similarly, NCCN guidelines^4^ for pancreatic cancer do not differentiate between invasive IPMN and PDAC. In modern times, high-volume centers for pancreatic surgery are oriented toward a neoadjuvant chemotherapy approach, as well as in resectable PDAC, but its use in invasive IPMN has not yet been investigated.

## Present

We performed an 11-year retrospective study^5^ in a high-volume center for pancreatic surgery that included patients who underwent resection for both de novo PDAC and invasive IPMN to assess both prognosis between the two adenocarcinoma subtypes and response to neoadjuvant therapy in both. Overall, our study confirmed that invasive IPMNs have a better prognosis when compared with de novo PDAC, with a 5-year overall survival of 52% versus 29% (*p* < 0.001), respectively. We found differences in the use of neoadjuvant chemotherapy between groups, with de novo PDAC having higher utilization (65%) than invasive IPMN (25%). However, invasive IPMN had similar neoadjuvant therapy response metrics to de novo PDAC as assessed by radiological response (RECIST criteria), tumor marker response (CA 19-9 normalization), metabolic response [fluorodeoxyglucose-positron emission tomography (FDG-PET)], and pathological treatment response (CAP score).

## Future

The results of our study further suggest that invasive IPMN likely has a more favorable biology when compared with de novo PDAC and a better overall prognosis. Despite the difference in oncological outcomes between the two pancreatic malignancies, it appears that the responses seen to neoadjuvant chemotherapy on de novo PDAC apply similarly to invasive IPMN, and thus can be used with less skepticism with the knowledge that the responses are comparable. However, preoperative chemotherapy in our cohort of invasive IPMN was used significantly less often than in the de novo PDAC cohort, suggesting that a neoadjuvant approach is still not as well accepted for invasive IPMN as it is for PDAC.

## Conflict of interest

No public or private funding was utilized in the development of this study. The authors have no conflicts of interest to declare.
